# Case Report: Somatic Symptoms Veiling Gender Dysphoria in an Adolescent

**DOI:** 10.3389/fped.2021.679004

**Published:** 2021-05-28

**Authors:** Giuliana Morabito, Dora Cosentini, Gianluca Tornese, Giulia Gortani, Serena Pastore, Maria Rita Lucia Genovese, Giorgio Cozzi

**Affiliations:** ^1^Department of Pediatrics, Santa Maria degli Angeli Hospital, Pordenone, Italy; ^2^Institute for Maternal and Child Health IRCCS “Burlo Garofolo”, Trieste, Italy; ^3^University of Trieste, Trieste, Italy

**Keywords:** mental health emergency, somatic symptom disorder, chronic pain, gender dysphoria, gender incongruence

## Abstract

**Background:** Somatic symptom disorder is common in children and adolescents; usually, it is an expression of a mental health problem or other conditions that lead to psychosocial impairment and suffering. Among these, in pubertal age, gender dysphoria should be considered.

**Case Presentation:** We present the case of a 15-year-old girl admitted to the hospital because of a 2-month history of scattered arthralgia and myalgia, headache, and fatigue, with repeated visits to the emergency room. The physical exam was unremarkable, except for step walking and pain. Repeated diagnostic tests were normal, and consecutive psychological interviews disclosed intense suffering due to a gender incongruence. Referral to the hospital gender service was offered and refused by the parents.

**Conclusions:** In pubertal age, gender dysphoria may be expressed through somatoform symptoms. Diagnosis is challenging to accept for the parents even in the presence of adequate multi-disciplinary hospital services.

## Introduction

Somatic symptoms are common in children and adolescents attending health services (1). When these cause significant distress and impairment of daily functioning (such as school absenteeism and avoidant behaviors), the condition is defined somatic symptom disorder (SSD) (2). Patients with SSD commonly seek help from different pediatric healthcare settings (3, 4), with pain as the most frequently reported physical symptom (5). A lot of external and individual factors, as well as psychiatric comorbidities, are associated with SSD (6); current research is exploring their role in the development of the disease, given the broad spectrum of the clinical and psychopathological pictures and the need to strengthen care and interventions (7). In this article, we present the case of a teenage girl who was admitted to the pediatric ward because of long-lasting somatic symptoms and finally revealing gender dysphoria.

## Case Report

A 15-year-old previously healthy girl was admitted to the pediatric ward because of a 2-month history of scattered arthralgia, myalgia, headache, and fatigue. The pain had started 2 weeks after a viral infection: initially, it was located to the wrists and then involved the thoracolumbar spine and both legs. The patient reported inconstant swelling and stiffness of the wrist, worsening of pain on contact with hot water, breaths, or body movements, partial response to physiotherapy, and progressive lacking or no improvement to NSAIDs. No trauma, fever, or weight loss were reported. Her medical history was remarkable for repeated visits to the Emergency Department (ED) owing to the symptoms: the results of the repeated diagnostic tests were all normal, including complete blood cell count, renal and hepatic function, serum electrolytes, total proteins, muscle enzymes (creatine kinase, aldolase), lactate dehydrogenase, uric acid, ESR and CRP, urinalysis. X-rays of the wrist and the column were both negative. Because of the appearance of step walking, she had been referred to the rheumatologic service of the hospital: a more extensive serological panel, including celiac serology, autoantibodies (ANA, dsDNA, ANCA, Lupus Anticoagulant), rheumatoid factor, Epstein-Barr virus, and Lyme-Borreliosis serology had yielded non-revealing findings. Antistreptolysin titer was slightly increased (638 UI/ml) with a regular pharyngeal swab. On her past history, 1 year before, she had performed blood exams because of a complaint of chronic fatigue.

Tests were all normal, scholar distress had emerged, and the symptoms were resolved after changing school and a psychotherapy session. She practiced rowing from the age of 10–13 years, then she did skiing and cheerleading, but the pain resulted in her abandoning her social and sports activities in the past month and missing 1 week of school. Due to the disturbing trend of the pain, with worsening of the gait and subsequent complaint of continuous night awakening, headache, and fatigue, the girl has been taken to the hospital for the third time and detained for observation. Her parents were concerned that this was a severe illness not adequately investigated, given the lack of response to drug treatment and the absence of a precise diagnosis. Physical examination on admission was unremarkable except for pain (rated six out of 10, worsening on movement) and a clumsy gait. The girl appeared smiling and collaborating with medical staff; vital signs were normal and cutaneous, cardiorespiratory, and abdominal examination. Joints movements were fluid without signs of arthritis. The girl also reported difficulty in concentrating, bewilderment with the “inability to put thoughts in order,” but during the medical examination, she was able to make conversation and answer appropriately. A fundus oculi and an MRI of the spine definitively ruled out the possibility of an organic disease. Concurrently, she underwent a neuropsychiatric consultation. At a psychological interview, difficulty in accessing and sharing the patient's authentic emotional states emerged.

Several private talks with the girl progressively disclosed a severe discomfort related to not feeling female. The girl reported that she had been struggling to stay with her body for 5 years; last year, she discovered she felt more gratified by being connoted with a different sex than the one assigned to birth. She revealed she had chosen for herself the name “Alex,” which could be interpreted both in male and in feminine meaning. She expressed a preference to be spoken without the use of feminine adjectives. She wore formal or sporty men's clothes and reported she did not want to use the skirt so that the inner thighs would not touch each other by touching the genitals. She reported being attracted by females and experienced difficulty because she could not externalize it. After discussing with parents about gender issues, somatic symptoms seemed to get improving, and the use of painkillers decreased. Referral to our hospital gender service was offered and refused by the parents. Seven months later, recalling the family, they informed us that those symptoms disappeared, the patient had started a new session of psychotherapy by its trusted specialist; she had cut her hair and had come out with some friends, asking to be called “Alex” and not to be spoken with feminine adjectives or pronouns.

**Figure 1:**
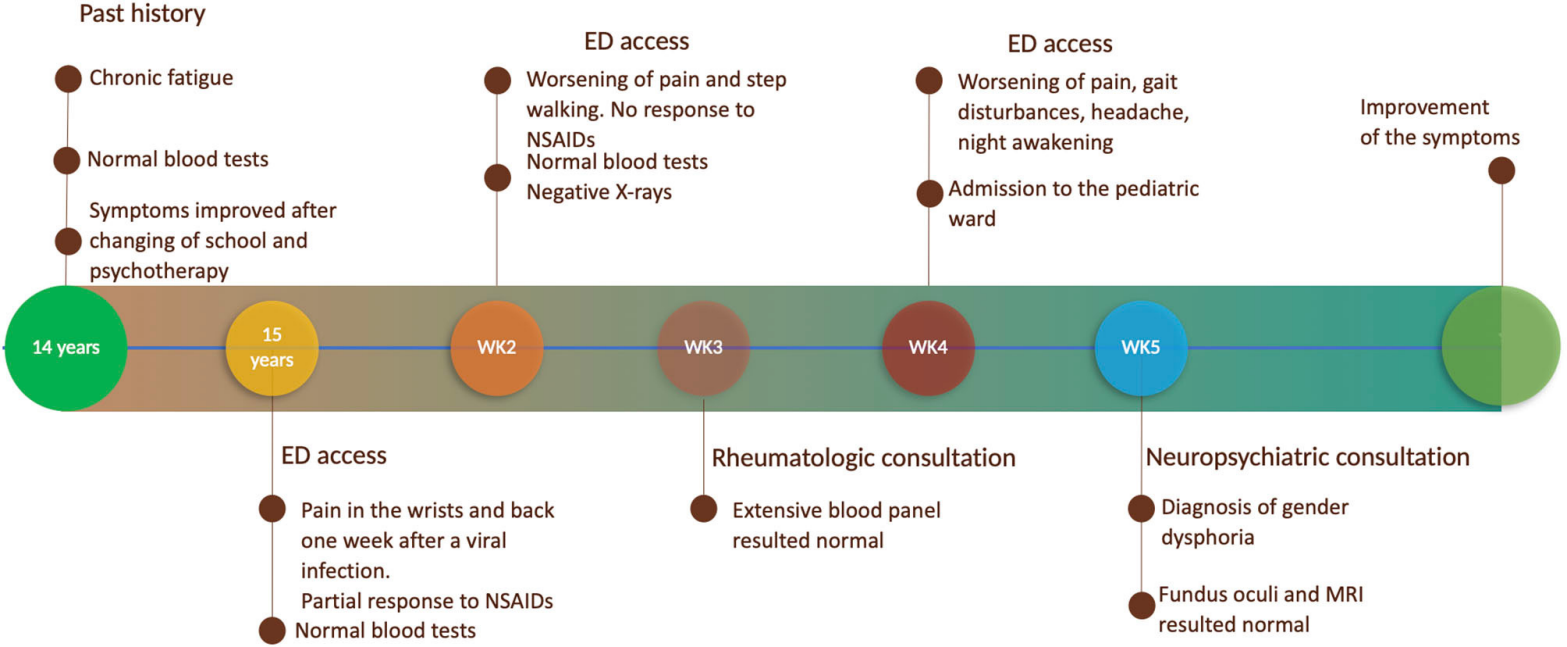
Timeline with relevant data about the episode of care.

Relevant data about the episode of care are summarized in Figure 1.

## Discussion

Gender Dysphoria (GD) is described in DSM-5 as the difficulty recognizing itself in the gender assigned at birth (8). This new definition differentiates from the previous “Gender Identity Disorder” in DSM-IV for the emphasis placed on the significant clinical suffering that scars the subject, with a functional, scholar, and social impairment associated with gender non-conformity. No exact data estimates the prevalence of gender dysphoria in children and adolescents due to the lack of representative studies and the definition “transgender” to indicate different entities (like gender dysphoria or gender non-conformity) (9). A recent review of 18 studies reported proportions of individuals with transgender and gender non-conforming-specific diagnoses ranging from 0.7 to 28 per 100,000 (10). A dramatically increasing number of referrals to gender clinics by adolescents with gender dysphoria, especially for native females, was reported (11), giving the impression of the phenomenon's dimension, mostly submerged. In line with DSM-5 criteria for GD, our patient showed a marked incongruence between the experienced gender and the assigned gender and a strong desire to be rid of her primary and secondary sex characteristics, and a strong desire to be treated as a different gender from the assigned one.

According to DSM-5, to diagnose GD, the symptoms described must be present for at least 6 months: in our patient, we can assume that the psychic suffering due to the incongruity between the gender experienced and that assigned at birth was present for much longer. In general, LGBT people make more use of mental health services and accident emergency services than the general population, according to a Scottish survey of 2012 (12). A strong correlation between GD and psychopathologies has already been described: Naata et al. (13) reported an association with psychological morbidity as depression (78.5%), anxiety (63.3%), PTSD (22.8%), eating disorders (12.7%), and bipolar disorder (5.1%) in transgender adolescents, with suicidal ideation (74.7%), and self-injurious behaviors (40–55%). Gender dysphoria-related suffering commonly emerges during adolescence with the development of secondary sexual characteristics.

Puberty is a critical time in which distress may intensify; gender dysphoria may emerge with the development of undesired secondary sex characteristics, as well as the likelihood of anxiety and depressive disorder (14). Nevertheless, these subjects may present psychological discomfort also in preadolescent age and may express this discomfort with physical symptoms. Although adolescent females more commonly report somatic symptoms, to our knowledge, there aren't specific symptoms complained more frequently by one particular gender.

Almost one-third of subjects have a history of at least one suicide attempt (15). Formerly the desire to change gender was interpreted as a symptom of psychopathology; recently, there is increasing evidence that mental health problems are the consequence of people's conflict with their biological gender, that emerges during growth and is associated with psychosocial distress (16). In this case, a history of long-lasting symptoms changing over time, with an unremarkable physical examination and repeated normal diagnostic tests, associated with disproportionate thoughts, feelings, and behaviors regarding the symptoms with disruption of everyday functioning, a repeated search for medical attention, and overwhelming concern of parents strongly suggested the hypothesis of a somatic symptoms disorder. Epidemiological studies show that children undergoing high levels of social or familial stress more frequently develop physical symptoms. We can't exclude that also in our case the fear for social or familial stigma related to the gender incongruence could have triggered or contributed to the development of somatic symptoms. Compared to DSM-5, the 6 months-duration criteria for the diagnosis of SSD was not attended. Literature shows that symptoms lasting 1 month and connected to psychosocial impairment in childhood or adolescence are highly suggestive of SSD (17); in our opinion, the severity of the whole picture, seen as the result of suffering in a context of vulnerability proper to the pediatric age, warrants immediate care.

Individual, familial, and social factors may interact in different ways to develop somatic symptom disorder (18–20). Association between GD and SSD has already been described (21). Children and adolescents often secretly hide their feelings related to gender non-conformity. In this context, physical symptoms may offer a means to express this distress and receive support. Another possible view is the somatic symptom as an expression of a poor bonding with one's unwanted body, which becomes a vehicle of malaise and suffering: 34.5% of adolescent transgender girls and 24.2% of transgender boys experience body dissatisfaction and body image problems (22). From our patient's perspective, the possibility to disclose her discomfort and to communicate her struggle for the desire to belong to the other sex seemed to open the way to face it as well as somatic symptoms. Recognizing people with GD is essential to offer proper intervention: literature shows a positive correlation between treatment and greater body satisfaction (23). It is demonstrated that social inclusion (including specific support from parents) affects reducing distress (i.e., rates of suicide ideation and attempts) (24). The strength of this case is to raise and discuss an issue that is frequently unrecognized in pediatric practice. Patients with gender dysphoria often have a significant burden of psychological suffering related to their condition; less frequently, this suffering is presented mainly with long-lasting physical symptoms. The main limit of this manuscript is that it refers to a single case, so that generalizations should be taken with caution.

## Conclusion

To our knowledge, this is the first pediatric case of gender dysphoria disclosed by a somatic symptom disorder. Diagnosis of GD is challenging to accept for the parents, even though adequate multi-disciplinary hospital services and the possibility of care are offered. GD is probably much more common than we assume, and it is associated with high-level suffering. As pediatricians, we should keep it in mind as a possible condition associated with long-lasting physical symptoms and functional impairment.

## Data Availability Statement

The original contributions presented in the study are included in the article, further inquiries can be directed to the corresponding author.

## Ethics Statement

Written informed consent was obtained from the minor's parents for the publication of any potentially identifiable images or data included in this article.

## Author Contributions

GM, DC, and GT conceptualized and drafted the initial manuscript and reviewed and revised the manuscript. GG, SP, and MG collected data and reviewed and revised the manuscript. GC conceptualized the manuscript, coordinated and supervised data collection, and critically reviewed the manuscript. All authors approved the final manuscript as submitted and agree to be accountable for all aspects of the work.

## Conflict of Interest

The authors declare that the research was conducted in the absence of any commercial or financial relationships that could be construed as a potential conflict of interest.
